# Drug utilisation and adverse drug reaction monitoring in schizophrenic patients: A tertiary care hospital study in India 

**DOI:** 10.6026/973206300223568

**Published:** 2026-06-30

**Authors:** Vipin Kumar Jain, Shilpa N Kaore, Megha Jain, Vaibhav Dubey

**Affiliations:** 1 Department of Pharmacology, Chhindwara Institute of Medical Sciences, Chhindwara, Madhya Pradesh, India; 2 Department of Pharmacology, All India Institute of Medical Sciences, Bhopal, Madhya Pradesh, India; 3 Department of Dentistry, Chhindwara Institute of Medical Sciences, Chhindwara, Madhya Pradesh, India; 4 Department of Psychiatry, PCMS & RC, Bhopal, Madhya Pradesh, India

**Keywords:** Drug utilization, adverse drug reaction (ADR), schizophrenia, tertiary hospital

## Abstract

Inadequate and poorly documented data on psychotropic drug utilization in schizophrenia in India necessitates systematic evaluation of prescribing patterns and associated adverse drug reactions. This prospective observational study assessed drug utilization, prescription rationality and adverse drug reactions among schizophrenia patients attending a tertiary care psychiatric outpatient department using WHO prescribing indicators. A total of 112 prescriptions comprising 285 drugs were analyzed, with atypical antipsychotics prescribed in 87% of first-visit cases, while combination therapies and polypharmacy were frequently observed. Significant dose variations were noted in commonly used antipsychotics such as risperidone, olanzapine and aripiprazole, as well as benzodiazepines like clonazepam and lorazepam (p<0.001). Atypical antipsychotics predominated due to better tolerability and lower extrapyramidal side effects, highlighting evolving prescribing trends. Thus, structured evidence on drug utilization patterns and variability in dosing, help in optimizing rational prescribing practices in schizophrenia management.

## Background:

Schizophrenia is a chronic and debilitating psychiatric disorder characterized by disturbances in thought processes, perception, emotional responsiveness and social functioning [1]. Psychosis, a hallmark feature of schizophrenia, results in a distorted or impaired sense of reality, often presenting as hallucinations, delusions and disorganized behaviour [2]. The etiology of schizophrenia is multifactorial, involving genetic, neurobiological and environmental components, which necessitates individualized and often complex pharmacological management strategies. Antipsychotic medications remain the cornerstone of treatment, broadly classified into typical (first-generation) and atypical (second-generation) agents, each differing in efficacy, safety profile and adverse effect spectrum [3]. The World Health Organization (WHO) defines drug utilization as the marketing, distribution, prescription and use of drugs in a society, with special emphasis on the resulting medical, social and economic consequences. Drug utilization studies are essential tools for evaluating prescribing patterns, identifying irrational drug use and assessing adherence to standard treatment guidelines [4]. Such studies also provide insight into the prevalence of polypharmacy, which is particularly common in psychiatric practice due to the need for symptom control, management of comorbidities and mitigation of adverse drug reactions. However, polypharmacy increases the risk of drug interactions, non-compliance and adverse effects, thereby impacting overall treatment outcomes [5]. In India, there is a paucity of systematically documented data regarding psychotropic drug utilization, especially in schizophrenia. Additionally, variations in prescribing practices across different healthcare settings and the lack of uniform adherence to international or national guidelines further complicate rational drug use [6]. Monitoring adverse drug reactions is equally critical, as antipsychotic medications are associated with significant side effects, including extrapyramidal symptoms, metabolic disturbances and sedation, which may affect patient compliance and quality of life [7]. Therefore, this study is important to assess the patterns of drug utilization, rationality of prescriptions and adverse drug reactions in schizophrenic patients in a tertiary care hospital setting.

## Methodology:

This observational, unicentric drug utilization study was conducted after obtaining approval from the Institutional Ethics Committee. The study was carried out in patients attending the Psychiatry Outpatient Department at PCMS & RC, Bhopal, Madhya Pradesh, over a period extending from December 2013 to May 2015. All patients diagnosed with schizophrenia, irrespective of gender, were included in the study provided they, or their legal guardians, were willing to give written informed consent and comply with regular follow-up for duration of four months. Patients such as infants, pregnant and lactating women and those suffering from epilepsy, mental retardation, senile degeneration, or dementia who were unable to comply with study requirements were excluded. Data collection was performed to evaluate multiple parameters, including drug utilization patterns, rationality of prescriptions and safety and efficacy outcomes. Drug utilization patterns were assessed in relation to demographic characteristics, the use of newer-generation psychotropic agents in place of conventional drugs, emerging indications for existing medications and the profile of co-prescribed drugs. The rationality of prescriptions was evaluated based on the number of drugs per prescription, appropriateness of indication, dose, frequency, duration of therapy, use of generic versus brand names, adequacy of patient information and concomitant medications. Safety and efficacy were monitored through adverse drug reaction (ADR) assessment conducted at monthly intervals throughout the follow-up period. The collected data were systematically compiled and organized into a master table, followed by meaningful categorization and presentation using tables and graphical representations. Statistical analysis was performed in two stages: Data compilation and presentation, followed by inferential statistical evaluation. The analysis was conducted using the Statistical Package for the Social Sciences (SPSS) version 20 (Chicago Inc., USA). Quantitative variables were expressed as mean values, while qualitative variables were presented as proportions. Appropriate statistical tests were applied to determine significance, with a p-value of less than 0.05 considered statistically significant.

## Results:

Table 1 presents the age and gender-wise distribution of schizophrenia patients (n=70), demonstrating a higher prevalence among males (41) compared to females (29). The majority of patients were concentrated in the 21-40 years age group, with 28 males and 16 females, indicating that schizophrenia predominantly affects individuals in early to middle adulthood, while very few cases were observed in the extreme age groups. Figure 1 (see PDF) illustrates the prescribing frequency of atypical antipsychotic drugs, highlighting their predominant use in clinical practice over conventional agents, which reflects a shift toward medications with improved safety and tolerability profiles. Table 2 depicts the distribution of the number of drugs per prescription (n=112), revealing that polypharmacy was common, with most prescriptions containing two (41%) or three drugs (38%). Monotherapy was relatively low (9%) and prescriptions with four or more drugs were less frequent, indicating a trend toward combination therapy in schizophrenia management. Figure 2 (see PDF) shows the mean doses of various drugs used in schizophrenia, including antipsychotics, antidepressants and benzodiazepines, indicating variability in dosing patterns across different drug classes, which may reflect individualized treatment approaches and clinical judgment. Table 3 summarizes the distribution of adverse drug reactions (ADRs) across different drug categories, classified into gastrointestinal (GIT), metabolic, autonomic nervous system (ANS) and central nervous system (CNS) effects. Olanzapine was associated with notable metabolic ADRs, while risperidone and clozapine showed a higher incidence of CNS-related effects. Most ADRs were mild to moderate in severity, indicating an overall manageable safety profile of the prescribed medications.

## Discussion:

In our study, male was more than female, similar to study of Hollingworth *et al.* (2010) [7], while another study by Alonso *et al.* (2004) [8] shown female preponderance. In current study prescriptions having two drugs were 41% accordance with findings of Cheekavolu *et al.* (2015) [9] in their study. We found that average number of drugs per prescription was 2.54 (285/112) although Nukala *et al.* (2019) [10] have reported it to be 1.19. In present study, the antipsychotics started in low doses and tailored according to response and ADRs. There is very high significant dose variation in Risperidone, high significant dose variation in olanzapine and aripiprazole in our study while in UK based study Harrington *et al.* (2002) [11] found that 20% cases received higher doses at initiation of therapy. In our study, greater burden of ADRs was shared by olanzapine, risperidone and aripiprazole and this was in accordance with the findings of study by Tripathi *et al.* (2022) [12]. Overall CNS and PNS related ADRs accounted for maximum ADRs compared to findings of Lucca *et al.* (2014) [13]. The co-prescribing of benzodiazepines (85%), anti-depressants (24%) and trihexyphenidyl (14%) was higher in our study compared to findings of Munjely *et al.* (2019) [14].

## Conclusion:

The study showed male predominance, with atypical antipsychotics most commonly prescribed, while typical agents were preferred in acute psychosis. Prescribing was largely rational with minimal polypharmacy and lower initial doses adjusted through a trial-and-error approach. CNS adverse drug reactions were most frequent and significant psychotropic use in younger patients remains a concern.

## Figures and Tables

**Table 1:** Age and Gender-wise distribution of patients of schizophrenia (n=70)

**Age group**	**Men (n=41)**	**Women (n=29)**
0-20	5	3
21-40	28	16
41-60	6	7
61-80	2	3
81-100	0	0

**Table 2:** Distribution of number of drugs per prescription

**No of drugs per prescription**	**No of prescriptions (112)**	**Percentage (%)**
1	10	9
2	46	41
3	43	38
4	12	11
5	0	0
6	1	0.9

**Table 3:** Distribution of various ADRs

**Drugs**	**GIT**	**Metabolic**	**ANS**	**CNS**
OLZ	5 (Mild) Moderat-8	11: Mild-3 ,	2 (Mild)	3 (Mild)
ARP	1(Mild)	-	-	1(Mild)
AMI	1(Mild)	-	-	1 (Mild)
QUT	-	-	-	1 (Mild)
RSP	-	-	-	11:Mild-4, Moderate -7
TRI	-	-	7; Mild-5, Moderate -2	
FLP	-	-	-	2 Moderate
FLZ	-	-	-	2:Mild-1, Moderate-1
ESC	2 (Mild)	-	-	-
FLU	2 (Mild)	-	-	-
SER	1 (Mild)	-	-	-
CLZ	-	-	-	6:Mild-2 Moderate-4
LRZ	-	-	-	3:Mild-2,Moderate-1
